# Human–Horse Neuromuscular Coordination Across Sequential Conditions During Physical Therapy Utilizing Equine Movement in Parkinson’s Disease

**DOI:** 10.3390/ani16213328

**Published:** 2026-10-22

**Authors:** Deanna M. Kennedy, Lucy Lai, Priscilla Lightsey, Nancy Krenek, Madison Weinrich, Rebecca M. Legere, Jennifer L. Zoller, Yiyu Wang, Renee Abbott, Taylor Nagle, Lynn Ruoff, Bob Byrns, Craig Huffhines

**Affiliations:** 1 Department of Kinesiology & Sport Management, Texas A&M University, College Station, TX 77843, USA; mdavis2798@tamu.edu (M.W.); taynagle18@tamu.edu (T.N.); 2 Institute for Equine Sciences, Texas A&M University, College Station, TX 77843, USA; priscilla@rockride.org (P.L.); nancy@rockride.org (N.K.); rlegere@cvm.tamu.edu (R.M.L.); jennifer.zoller@ag.tamu.edu (J.L.Z.); craig.huffhines@ag.tamu.edu (C.H.); 3 Theorem B, Bryan, TX 77802, USA; lucy@theoremb.com; 4 Department of Large Animal Clinical Sciences, Texas A&M University, College Station, TX 77843, USA; 5 Department of Agricultural Leadership Education & Communication, Texas A&M University, College Station, TX 77843, USA; 6 Department of Psychology, Princeton University, Princeton, NJ 08544, USA; yw4722@princeton.edu; 7 Department of Aerospace Engineering, Texas A&M University, College Station, TX 77843, USA; renee.abbott@tamu.edu; 8 Department of Veterinary Integrative Biosciences, Texas A&M University, College Station, TX 77843, USA

**Keywords:** human-animal interaction, Parkinson’s disease, physical therapy utilizing equine movement, hippotherapy, electromyography, human-horse coordination, postural control

## Abstract

This exploratory, single-session, repeated-measures study characterized human and equine trunk muscle activity and human–horse EMG covariation during physical therapy utilizing equine movement in individuals with Parkinson’s disease (PD). Nine individuals with PD and three horses completed pre-session quiet sitting (QS Pre), figure-eight riding (F8), stop-and-go riding (SNG), and post-session quiet sitting (QS Post). Bilateral surface EMG was recorded from three human and four equine trunk muscles. All analyses were exploratory and examined four outcomes: normalized EMG amplitude, zero-lag human–horse correlation, lagged human–horse correlation, and human–horse EMG-EMG coherence. Human normalized EMG amplitude did not differ across QS Pre, F8, and SNG. Equine amplitude was greater during F8 than QS Pre (difference 9.43 percentage points, 95% CI [4.27, 14.59]). SNG also showed greater amplitude than QS Pre (5.30 percentage points, 95% CI [0.32, 10.27]), although this difference was not statistically significant in the participant-level sensitivity analysis. F8 exceeded SNG in the direct comparison (4.43 percentage points, 95% CI [1.44, 7.41]) but not in the adjusted three-condition comparison. Zero-lag correlations were generally close to zero; SNG exceeded F8 by only 0.0056 Fisher’s z units (95% CI [0.0004, 0.0107]) in the direct comparison, but this difference was not statistically significant in the adjusted three-condition comparison. When temporal offsets of ±300 ms were permitted, lagged correlation strength was greater during F8 than QS Pre (difference 0.013, 95% CI [0.003, 0.024]) and during SNG than QS Pre (0.011, 95% CI [0.001, 0.021]), with no difference between riding conditions. Median wavelet coherence was greater during QS Pre than F8 (difference 0.016, 95% CI [0.013, 0.018]) or SNG (0.013, 95% CI [0.011, 0.015]), while SNG exceeded F8 overall (0.003, 95% CI [0.002, 0.004]). Simultaneous human and equine EMG was feasible during the sequential mounted conditions, but the observed signal properties differed across outcomes. Because the conditions were administered in a fixed sequence, their individual effects could not be distinguished from order-related influences. These exploratory findings require confirmation in larger studies with counterbalanced task orders, controlled gait characteristics, and relevant clinical outcomes.

## Simple Summary

Physical therapy utilizing equine movement may help people with Parkinson’s disease, but little is known about how muscle activity in the rider and horse changes during different mounted tasks. This exploratory, single-session study simultaneously recorded trunk muscle activity from nine individuals with Parkinson’s disease and three therapy horses during sequentially administered conditions involving quiet sitting, figure-eight riding, and stop-and-go riding. Human muscle activity remained relatively stable across the riding tasks, while horse muscle activity showed clearer differences between conditions. Relationships between human and horse muscle signals also varied depending on whether activity was examined at the same moment, with short time delays, or by frequency-specific patterns. Importantly, these measures did not all change in the same direction, and the fixed task order prevented the effects of individual activities from being separated from changes occurring over the session. The findings should not be interpreted as evidence of therapeutic effectiveness or direct physiological coupling between rider and horse. The study shows that simultaneous recording of human and horse muscle activity is feasible during physical therapy utilizing equine movement. Larger studies with counterbalanced task order, better control of horse movement, and clinical outcome measures are needed to determine the relevance of these patterns.

## 1. Introduction

Physical therapy (PT) utilizing equine movement incorporates the motion of a horse as a treatment tool to address functional impairments. It falls within the broader umbrella of equine-assisted services (EAS). Hippotherapy refers to the purposeful use of equine movement within occupational therapy, PT, or speech-language pathology. During walking, the horse generates rhythmic, multidirectional movements that challenge the rider’s postural control and require ongoing trunk regulation [1,2,3]. Maintaining postural stability involves integrating visual, vestibular, and somatosensory information with coordinated activation of the trunk muscles [4,5]. These demands make equine movement relevant to rehabilitation in populations with impaired postural control, including individuals with Parkinson’s disease (PD). PD commonly impairs axial motor control and postural stability, contributing to gait limitations, falls, and functional disability [6,7,8,9,10,11,12,13]. Studies involving live horses or horse simulators have reported functional benefits in individuals with PD [14,15,16], but the neuromuscular demands of this treatment context remain largely unexplored.

During mounted locomotion, horse movement provides a dynamic support surface that elicits ongoing postural adjustments by the rider [17], while rider mass and posture influence the magnitude and distribution of forces transmitted to the horse [18,19]. This mechanical interface provides a rationale for examining associations between rider and equine muscle activity. Coordination dynamics provides a framework for considering how interacting systems organize their behavior [20,21,22], while Newell’s constraints model emphasizes the contributions of organism, task, and environmental constraints [23]. Previous horse–rider studies have demonstrated movement coordination using relative phase, temporal synchronization, and kinematic cross-correlation [24,25,26,27]. In parallel, rider electromyography (EMG) studies in older adults and children with cerebral palsy have characterized trunk activation during EAS interventions [28,29], while equine EMG studies have characterized muscle activation during locomotion, exercise, and clinically relevant movement tasks [30,31,32].

Simultaneous EMG permits individual muscle responses and associations across the dyad to be examined within the same task [33,34]. Normalized EMG amplitude characterizes activation magnitude relative to a muscle-specific reference [35,36]. Zero-lag correlation describes concurrent covariation in EMG envelopes, whereas lagged correlation evaluates covariation when temporal offsets are allowed [37,38]. These offsets may be relevant because mechanical transmission and sensorimotor responses need not occur simultaneously [39,40]. EMG-EMG coherence complements the time-domain analyses by quantifying frequency-specific linear association between signals [41,42,43]. Together, these measures characterize activation magnitude, concurrent and time-shifted covariation, and shared spectral structure. Within the constraints framework [23], they provide complementary ways to characterize individual muscle activity and its organization across the human–horse dyad under different mounted conditions.

Accordingly, this study simultaneously recorded EMG from individuals with PD and horses during a single PT session utilizing equine movement to examine normalized muscle activity and temporal and frequency-domain associations across the human–horse dyad. Individuals with PD were examined because impaired axial and postural control makes the neuromuscular demands of this treatment context clinically relevant [9,12,13]. Conditions comprised quiet sitting (QS) on a stationary horse, continuous figure-8 riding (F8), and stop-and-go riding (SNG). QS provided a stationary mounted reference that retained the seated posture and horse–rider interface while largely removing locomotor perturbations. F8 and SNG differed in their movement characteristics, with continuous locomotion during F8 and repeated stopping and restarting during SNG, which also incorporated verbal commands and ring handling. Guided by the theoretical framework, we expected greater normalized rider trunk EMG amplitude and stronger human–horse EMG associations during riding than during QS because mounted locomotion introduces additional postural demands and requires ongoing rider adjustments to the horse’s rhythmic movement [24,25]. Because the conditions were administered in a fixed sequence, the comparisons were intended to characterize differences across conditions rather than isolate the independent effects of specific task characteristics. These comparisons were exploratory, as were differences between F8 and SNG, individual human and equine muscle responses, muscle pair associations, lag characteristics, and frequency-specific coherence.

## 2. Methods

### 2.1. Participants

Nine older adults with PD (mean age = 73.9 ± 5.7 years; 5 males and 4 females) participated in this study. Participants were recruited through the Robert Conte Foundation for Parkinson’s Disease and Movement Disorders. All participants were assessed during their usual ON-medication state. Participant characteristics are summarized in Table 1. Three highly trained horses (two unregistered Quarter Horse geldings and one unregistered Quarter Horse mare; ages = 15, 16, and 21 years) from Texas A&M University Courtney Grimshaw Equine-Assisted Services Program (Courtney Cares), a Professional Association of Therapeutic Horsemanship (PATH) International Premier Accredited Center, participated in the study. The horses were selected based on temperament, consistency of gait, soundness, and prior therapeutic riding experience [44,45]. Human participants were eligible if they were 18 to 85 years of age, weighed no more than 200 lb. (90.7 kg), had a medical diagnosis of PD, were able to safely participate in the study, and had no medication changes within the previous three weeks. Exclusion criteria included a history of stroke or traumatic brain injury, implanted electronic devices (e.g., pacemakers or deep brain stimulators), allergy to silver electrodes, damaged skin at EMG electrode placement sites, medical or orthopedic conditions that precluded safe horseback riding, significant cognitive impairment based on the Mini-Mental State Examination (MMSE), or other conditions that prevented safe participation.

No a priori power calculation was performed. The sample size reflected the routine clinical configuration at the facility, where three horse–rider dyads participate per PT session. Data were collected across three sessions conducted on the same day, resulting in nine participants paired with three therapy horses. The study was approved by the Texas A&M University Institutional Review Board (IRB2022-1504F) for human subjects and the Institutional Animal Care and Use Committee (IACUC2022-0262) for animal procedures. All human participants provided written informed consent before participation.

### 2.2. Experimental Protocol

**Figure 1:**
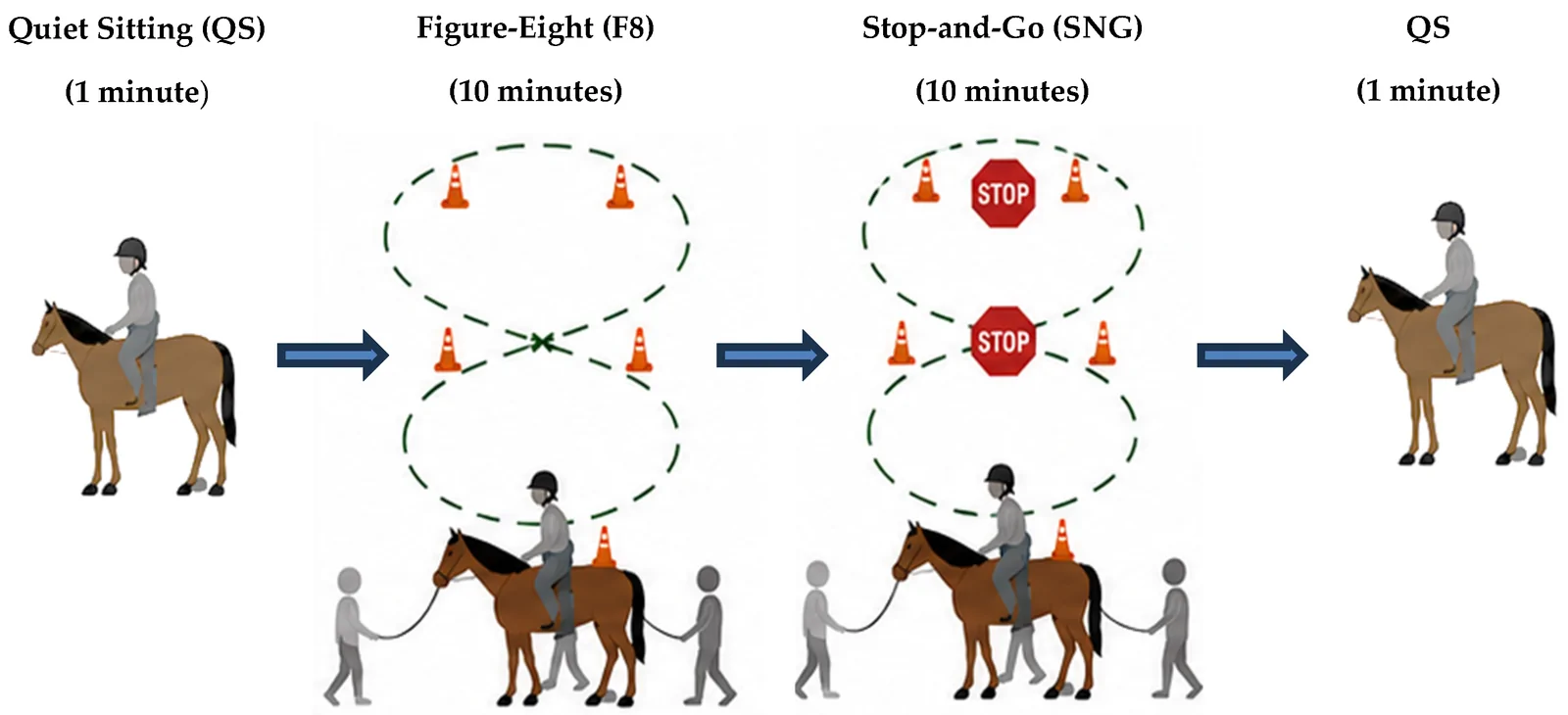
Experimental protocol. Participants first completed a 1 min quiet sitting (QS Pre) condition while seated on a stationary horse, followed by 10 min of figure-8 riding (F8) and 10 min of stop-and-go riding (SNG). Following the riding tasks, participants completed a second 1-min quiet sitting (QS Post) condition. A horse handler and two trained side-walkers accompanied each participant throughout all mounted activities to ensure safety.

This exploratory, single-session, repeated-measures study was designed to characterize human and equine muscle activity and human–horse neuromuscular coordination during PT utilizing equine movement in individuals with PD. It was not designed to evaluate the clinical effectiveness of the intervention. Data collection was conducted across three testing sessions, with three human–horse dyads participating simultaneously during each session (Figure 1). The same three horses participated in all sessions, resulting in a total of nine unique human–horse dyads. Each participant remained paired with the same horse throughout the experimental session to maintain a consistent human–horse pairing.

The intervention was conducted at Courtney Cares under the supervision of a licensed physical therapist who was both a Hippotherapy Clinical Specialist and PATH International Certified Therapeutic Riding Instructor (CTRI). Participants wore an American Society for Testing and Materials (ASTM) International/Safety Equipment Institute (SEI)-approved equestrian riding helmet and a gait belt throughout the intervention. Horses were equipped with a saddle pad, bareback pad, girth, and side-pull. Stirrups were omitted to reduce lower-extremity support, thereby increasing the postural demands placed on the trunk during the riding experience. Each participant was accompanied by one horse handler and two trained side-walkers to ensure safety during mounting, riding, and dismounting.

Participants first completed a one-minute pre-session quiet sitting condition (QS Pre), during which the horse remained stationary while the participant sat quietly on the horse. The PT session utilizing equine movement consisted of approximately 20 min of riding activities performed at the walk and was divided into two consecutive tasks. During the first 10 min, participants continuously rode a standardized figure-of-eight (F8) pattern marked with traffic cones. During the second 10 min, participants completed the same F8 pattern while performing repeated stop-and-go (SNG) transitions at approximately one-minute intervals (Figure 1). Participants held a rubber ring with both hands during the SNG task. During SNG, the physical therapist verbally delivered the command “whoa” to cue each stop. The horses were trained to respond to this auditory cue, while the horse handler simultaneously stopped walking. Side-walkers remained positioned alongside the participants throughout the task to provide safety support as needed. Horse walking speed and cadence were not experimentally standardized. Immediately following the riding tasks, participants completed a second one-minute post-session quiet sitting condition (QS Post) using the same standardized procedures. The mounted activities followed the routine clinical sequence used at the facility, with F8 preceding SNG for all participants. Accordingly, task order was not counterbalanced, and the protocol was designed to characterize neuromuscular behavior during a naturalistic clinical treatment sequence rather than to isolate task effects under counterbalanced experimental conditions.

Surface EMG was recorded continuously from both the participant and horse during QS Pre, F8, SNG, and QS Post. During the riding tasks, each completed figure-eight circuit was identified during data collection by an event marker inserted as the horse passed a predefined cone adjacent to the experimenter. These event markers were subsequently used during data extraction to identify the approximate starting locations of individual riding trials, with synchronized video used to verify the corresponding task events and analysis segments. For SNG, each stop event served as the reference point for a 32 s analysis window comprising the 16 s preceding and 16 s following the stop. For F8, corresponding 32 s windows were extracted from the equivalent location along the riding course to provide comparable movement sequences between tasks. Depending on individual riding speed, participants completed approximately 5 to 9 analyzable riding trials during each 10-min riding task.

### 2.3. Human and Equine Electromyography

Surface EMG was recorded simultaneously from the bilateral human erector spinae (ES), latissimus dorsi (LD), and rectus abdominis (RA) muscles and the bilateral equine gluteus medius (GM), latissimus dorsi (LD), rectus abdominis (RA), and splenius (SP) muscles using Trigno Centro 16-Avanti wireless EMG systems (Delsys Inc., Natick, MA, USA) sampled at 1000 Hz. Left- and right-sided muscles were recorded as separate channels and retained as distinct channels throughout signal processing and the analyses.

Human electrodes were placed according to the Surface Electromyography for the Non-Invasive Assessment of Muscles (SENIAM) recommendations. For the horses, muscle bellies were identified using diagnostic ultrasound to facilitate consistent electrode placement, and surface electrodes were placed by a board-certified large animal internal medicine specialist (DACVIM-LA) following standardized equine surface EMG procedures. Delsys Adhesive Sensor Interfaces were used to secure the sensors to the skin in both humans and horses. For equine recordings, veterinary tissue adhesive was additionally applied around the outer plastic rim of the sensor pod to improve fixation in the presence of sweat. For human recordings, adhesive skin wrap was used to further secure the sensors, minimize movement-related artifacts, and protect the electrode sites during mounted activity.

Three independent wireless acquisition systems were used to permit simultaneous recording from the three human–horse dyads participating in each PT session. Each acquisition system recorded EMG signals from one human–horse dyad. The three wireless systems were configured to operate on separate transmission frequencies to minimize radio frequency interference between systems during simultaneous data collection. For each system, data were wirelessly transmitted from the Trigno Avanti sensors to the Trigno Centro base station, which sent analog signals through Trigno Sensor Interfaces to a USB I/O device (National Instruments USB-6210, Austin, TX, USA) connected to a laptop. A custom LabVIEW (2020) program installed on all three laptops was used for data acquisition.

### 2.4. EMG Signal Processing

EMG signals were processed offline using custom MATLAB routines (R2024b: MathWorks, Natick, MA, USA). Signals underwent DC offset removal and fourth-order zero-phase Butterworth band-pass filtering (20–450 Hz). Powerline interference was evaluated independently for each trial and EMG channel using Welch power spectral density estimation, with a 1 s window and 50% overlap. A 60 Hz spectral prominence ratio was calculated as the mean power spectral density from 59 to 61 Hz divided by the mean power spectral density from 55 to 65 Hz, excluding 59–61 Hz. A predefined processing threshold of 2.0 was used to identify recordings with prominent 60 Hz interference. Because notch filtering can attenuate both powerline interference and physiological EMG content at the affected frequency [46,47,48], a 60 Hz notch filter (Q = 30) was applied using zero-phase filtering only when this threshold was exceeded. Signals were then full-wave rectified. Normalized EMG amplitude was calculated from the rectified signal. For zero-lag and lagged correlation analyses, the rectified signal was additionally low-pass-filtered using a fourth-order zero-phase Butterworth filter at 6 Hz to generate a linear envelope. EMG-EMG coherence was calculated from the rectified EMG signal using wavelet-based procedures [41,42].

Digital event markers inserted during data collection and verified using synchronized GoPro video (GoPro, Inc., San Mateo, CA, USA) were used to identify corresponding analysis segments across conditions. For the QS conditions, the 60 s EMG recording and synchronized video were visually inspected to identify a 32 s period during which both the horse and participant remained stationary. The selected QS segment was subsequently processed using the same procedures as the F8 and SNG analysis windows. For the SNG task, each stop event served as the reference point, and a 32 s analysis window consisting of the 16 s preceding and the 16 s following the stop was extracted. For the F8 task, corresponding 32 s windows were extracted from the equivalent location along the riding course to ensure comparable movement sequences between tasks. Because F8 involved continuous walking, whereas SNG included stopping and restarting, the 32 s windows represented the complete movement sequences associated with each condition rather than matched periods of uninterrupted locomotion. Accordingly, differences in the proportion of time spent walking and stationary were retained as characteristics of the recorded conditions.

For zero-lag and lagged correlation analyses, the first and last second of each analysis window were removed before feature extraction to minimize edge effects associated with filtering [49]. For EMG-EMG coherence analyses, edge effects were addressed by excluding time-frequency points outside the cone of influence [50].

All processed recordings underwent a predefined quality control (QC) procedure evaluating signal clipping, transient spikes, signal dropout, low-frequency contamination, 60 Hz powerline interference, high-frequency noise, and abnormal signal amplitude. These criteria were used to identify potential acquisition and movement-related contamination during mounted activity. Channel-trial records failing QC were excluded from downstream analyses. Trials that were unavailable in the source data, including recordings lost because of a data-saving failure for one participant, were treated as missing rather than as QC failures. Supplementary Table S1 summarizes data availability, QC exclusions and their reasons, condition-specific retention rates, and the number of valid observations contributing to each mixed-effects model.

### 2.5. Outcome Measures

The selected EMG outcomes were chosen to characterize complementary aspects of neuromuscular activity and human–horse coordination. Normalized EMG amplitude quantified within-muscle signal magnitude relative to a participant- and channel-specific reference. Zero-lag correlation quantified simultaneous human–horse EMG covariation, whereas lagged correlation quantified coupling strength allowing for temporal offsets between signals. EMG-EMG coherence quantified frequency-specific shared oscillatory activity between human and equine muscles. Outcome measures were calculated following signal preprocessing and QC.

### 2.6. Normalized EMG Amplitude

Mean rectified EMG amplitude was calculated from the full-wave rectified EMG signal within each analysis window. To account for differences in signal scale across participants, muscles, and species, amplitude was normalized using a participant and channel-specific reference value defined as the 95th percentile (P95) of rectified EMG values pooled across all QC-approved trials and conditions for that channel (Supplementary Table S1). Trial-level mean amplitude was expressed as a percentage of P95 [35,36]. Because surface EMG amplitude is influenced by muscle, anatomical, and recording-specific factors, normalization was used to support within-channel comparisons across experimental conditions [35]. Accordingly, absolute amplitude differences across muscles or species were not interpreted as differences in relative physiological recruitment. Rather, muscle effects were used to characterize the distribution of recorded EMG signal magnitude across the measured muscles, whereas condition effects were used to evaluate differences in signal magnitude across the sequentially administered conditions.

### 2.7. Zero-Lag Human–Horse EMG Correlation

Zero-lag temporal covariation between human and equine muscle activity was quantified using the Pearson correlation between paired rectified EMG linear envelopes. Correlation coefficients (r) were transformed using Fisher’s z transformation before statistical analysis to improve distributional properties. Positive values indicated positive simultaneous covariation between human and equine EMG signals, values near zero indicated little or no zero-lag linear association, and negative values indicated inverse covariation. Because locomotor EMG contains gait-related rhythmic structure, simultaneous covariation between EMG signals may reflect shared movement-related activation patterns rather than direct physiological coupling [51,52]. Accordingly, zero-lag correlation was interpreted as a measure of simultaneous temporal covariation rather than as evidence of direct physiological coupling.

### 2.8. Lagged Human–Horse EMG Correlation

To characterize human–horse EMG covariation while allowing for temporal offsets between signals, cross-correlation was calculated across a prespecified ±300 ms lag window [37,38]. Allowing temporal offsets was considered relevant because mechanically mediated interactions and sensorimotor responses need not occur simultaneously [39,40]. For each human–horse muscle pair and trial, the maximum absolute cross-correlation coefficient within the prespecified lag window was identified as the lagged-correlation measure. Because lagged associations between rhythmic signals do not establish the direction or mechanism of interaction, they were interpreted as temporal covariation rather than evidence of causal physiological coupling [38,40]. A sensitivity analysis expanded the lag-search window to ±500 ms.

### 2.9. Human–Horse EMG-EMG Coherence

Frequency-specific covariation between human and horse EMG signals was quantified using Morlet wavelet coherence applied to the rectified EMG signals [41,50,53]. Wavelet coherence provides a time-frequency representation of the association between paired signals, with values ranging from 0 to 1 [50]. Coherence was evaluated for each human–horse muscle pair within four prespecified frequency bands: alpha (5–13 Hz), beta (13–30 Hz), low gamma (30–60 Hz), and high gamma (60–100 Hz). These bands were selected based on commonly used frequency ranges in human EMG coherence research [33,34,42,54,55] and were applied identically to human and equine signals to provide a consistent framework for exploratory cross-species comparison. Because corresponding physiological frequency-band classifications have not been established for equine EMG, the frequency-band labels were used descriptively and were not assumed to represent identical physiological mechanisms across species.

Wavelet parameters were set to dj = 1/8, J1 = 64, and s0 = 2 dt, using a Morlet wavelet parameter of 6. Wavelet coherence was calculated using Gaussian smoothing in time and boxcar smoothing across scales (scale-smoothing width parameter = 0.6), following the Grinsted wavelet coherence implementation [50]. Time-frequency points outside the cone of influence were excluded to minimize edge effects [53]. Background coherence significance thresholds were estimated separately for each EMG pair and trial using 300 Monte Carlo realizations of independent autoregressive (AR(1)) surrogate signal pairs. Scale-specific thresholds were defined by the 95th percentile of the surrogate coherence distributions, considering only time-frequency points within the cone of influence [50]. Median coherence within each frequency band was calculated across all finite time-frequency coherence values within the band and inside the cone of influence [50]. Surrogate-derived thresholds were used to identify time-frequency points with coherence exceeding the estimated background level. However, median coherence was calculated from all valid time-frequency points within each frequency band, regardless of statistical significance. Coherence was interpreted as frequency-specific covariation between human and equine muscle activity rather than evidence of direct or causal physiological coupling.

Wavelet coherence was calculated using Gaussian smoothing in time and boxcar smoothing across scales (scale-smoothing width parameter = 0.6), following the Grinsted wavelet coherence implementation. Background coherence significance thresholds were estimated separately for each EMG pair and trial using 300 Monte Carlo realizations of independent AR(1) surrogate signal pairs. Scale-specific thresholds were defined by the 95th percentile of the surrogate coherence distributions, with time-frequency points outside the cone of influence excluded. These thresholds were used to calculate supplementary coherence significance metrics but were not applied to the median coherence values used in the mixed-effects analyses.

### 2.10. Statistical Analysis

Linear mixed-effects models were used to account for repeated observations within participants and muscle pairs, unequal numbers of available observations across participants, and the inclusion of all available data [56]. Across outcomes, analyses were structured to address three questions: (1) whether measures changed from pre- to post-session quiet sitting, (2) whether measures differed between the sequentially administered riding conditions, and (3) how the riding conditions compared with pre-session quiet sitting. The two-condition models provided direct comparisons between the corresponding conditions, whereas the three-condition models evaluated both riding conditions relative to QS Pre and included Bonferroni-adjusted pairwise comparisons among all three conditions. Because the models differed in the conditions included and the number of comparisons evaluated, their estimates and statistical significance were interpreted within their respective analytical frameworks.

For normalized EMG amplitude, human and equine data were analyzed separately. Fixed effects included Condition, Muscle, and the Condition × Muscle interaction. Human muscles included the erector spinae (ES), latissimus dorsi (LD), and rectus abdominis (RA); equine muscles included the splenius (SP), latissimus dorsi (LD), gluteus medius (GM), and rectus abdominis (RA). Repeated condition × muscle observations within participants were modeled using a compound symmetry covariance structure. Separate analyses were conducted for comparison of QS Pre with QS Post, comparison of F8 with SNG, and comparison of F8 and SNG with QS Pre. As a sensitivity analysis, normalized amplitude was averaged across muscles within each participant and condition, and condition effects were re-evaluated using participant-level observations.

For zero-lag human–horse EMG correlation, the dependent variable was the Fisher’s z-transformed signed Pearson correlation between human and equine EMG linear envelopes at zero lag. Fisher’s z transformation was applied to each eligible trial-level correlation before averaging across trials within each participant, condition, and side-specific human–horse muscle pair. Each observation in the statistical dataset therefore represented one participant × condition × side-specific muscle pair combination. Condition was included as a fixed effect. Participant and muscle pair were included as random intercepts, and repeated observations across condition were modeled within participant × muscle-pair combinations using an unstructured covariance structure. For lagged human–horse EMG correlation, the primary dependent variable was the absolute correlation strength obtained within a prespecified ±300 ms lag-search window. The maximum absolute correlation was identified separately for each eligible trial and subsequently averaged across trials within the same participant, condition, and side-specific muscle pair. The same mixed-model structure used for the zero-lag correlation analysis was applied. Sensitivity analyses evaluated the influence of the largest positive residual in the three-condition model and the effect of expanding the lag-search window from ±300 to ±500 ms.

For human–horse EMG coherence, the dependent variable was median wavelet coherence within four frequency bands: alpha (5–13 Hz), beta (13–30 Hz), low gamma (30–60 Hz), and high gamma (60–100 Hz). Trial-level median coherence values were averaged across eligible trials within each participant, condition, side-specific human–horse muscle pair, and frequency band before statistical modeling. Each observation in the analysis dataset therefore represented one participant × condition × side-specific muscle pair × frequency band combination. Fixed effects included Condition, Frequency Band, and the Condition × Frequency Band interaction. Participant and muscle pair were included as random intercepts, and repeated condition × frequency band observations were modeled within participant × muscle pair combinations using an unstructured covariance structure. Significant interactions were examined using within-band comparisons of estimated marginal means. Expanded random effects sensitivity models were also attempted for the three-condition comparison (QS Pre, F8, and SNG), incorporating participant-specific frequency-band and condition effects. These models did not achieve convergence. Therefore, their estimates were not interpreted or reported.

Models were estimated using restricted maximum likelihood (REML), with the Kenward–Roger approximation used to estimate denominator degrees of freedom. Estimated marginal means and 95% confidence intervals were obtained from the fitted models. Pairwise and post hoc comparisons were adjusted using the Bonferroni procedure where applicable. Model assumptions were evaluated by inspection of residual distributions, Q-Q plots, and residual-versus-predicted plots, with targeted sensitivity analyses performed when influential observations or notable departures from assumptions were identified. All statistical analyses were conducted using IBM SPSS Statistics (Version 31; IBM Corp., Armonk, NY, USA), with statistical significance set at α = 0.05.

## 3. Results

Of 2296 available channel-trial records evaluated during QC, 2187 (95.3%) were retained and 109 (4.7%) were excluded because of clipping (*n* = 97) or high-frequency contamination (*n* = 12). Additional channel-trial entries were unavailable because the corresponding trials were not present in the source data. For one participant, this was due to a data saving failure. These entries were treated as missing rather than as QC exclusions. No complete available experimental trial was excluded because of QC failure. QC accounting by task is provided in Supplementary Table S1.

### 3.1. EMG Amplitude

#### Quiet Sitting Before and After the Session

Human normalized EMG amplitude was lower at QS Post (estimated marginal mean ± SE: 27.50 ± 2.67% of P95) than at QS Pre (34.94 ± 2.49% of P95), although the pairwise difference was not statistically significant (QS Post–QS Pre: −7.43 percentage points, 95% CI [−15.02, 0.15], *p* = 0.055) (Table 2). Equine normalized amplitude was lower at QS Post (19.61 ± 1.97% of P95) than at QS Pre (25.39 ± 1.85% of P95; difference = −5.77 percentage points, 95% CI [−10.70, −0.85], *p* = 0.022).

### 3.2. Comparison Between Sequential Riding Conditions

In the separate two-condition analysis, human normalized EMG amplitude did not differ significantly between F8 and SNG, F(1, 39.50) = 0.18, *p* = 0.674. Estimated marginal means were 37.57% of P95 for F8 and 36.76% for SNG (F8—SNG: 0.81 percentage points, 95% CI [−3.27, 4.89], pairwise *p* = 0.690) (Table 2). Neither the muscle effect, F(2, 37.72) = 1.07, *p* = 0.352, nor the condition × muscle interaction, F(2, 37.72) = 0.32, *p* = 0.731, was significant. Equine normalized EMG amplitude was greater during F8 than SNG, F(1, 53.80) = 9.47, *p* = 0.003. Estimated marginal means were 35.11% of P95 for F8 and 30.68% for SNG (F8—SNG: 4.43 percentage points, 95% CI [1.44, 7.41], pairwise *p* = 0.004). The muscle effect was significant, F(3, 51.93) = 4.72, *p* = 0.006, whereas the condition × muscle interaction was nonsignificant, F(3, 51.93) = 0.53, *p* = 0.662. Participant-level sensitivity analyses retained the nonsignificant human condition effect, F(1, 8.15) = 0.20, *p* = 0.667, and the significant equine condition effect, F(1, 7.22) = 17.52, *p* = 0.004. The equine F8—SNG difference was 4.40 percentage points (95% CI [0.73, 8.08], pairwise *p* = 0.025).

### 3.3. Riding Conditions Compared with Pre-Session Quiet Sitting

**Figure 2:**
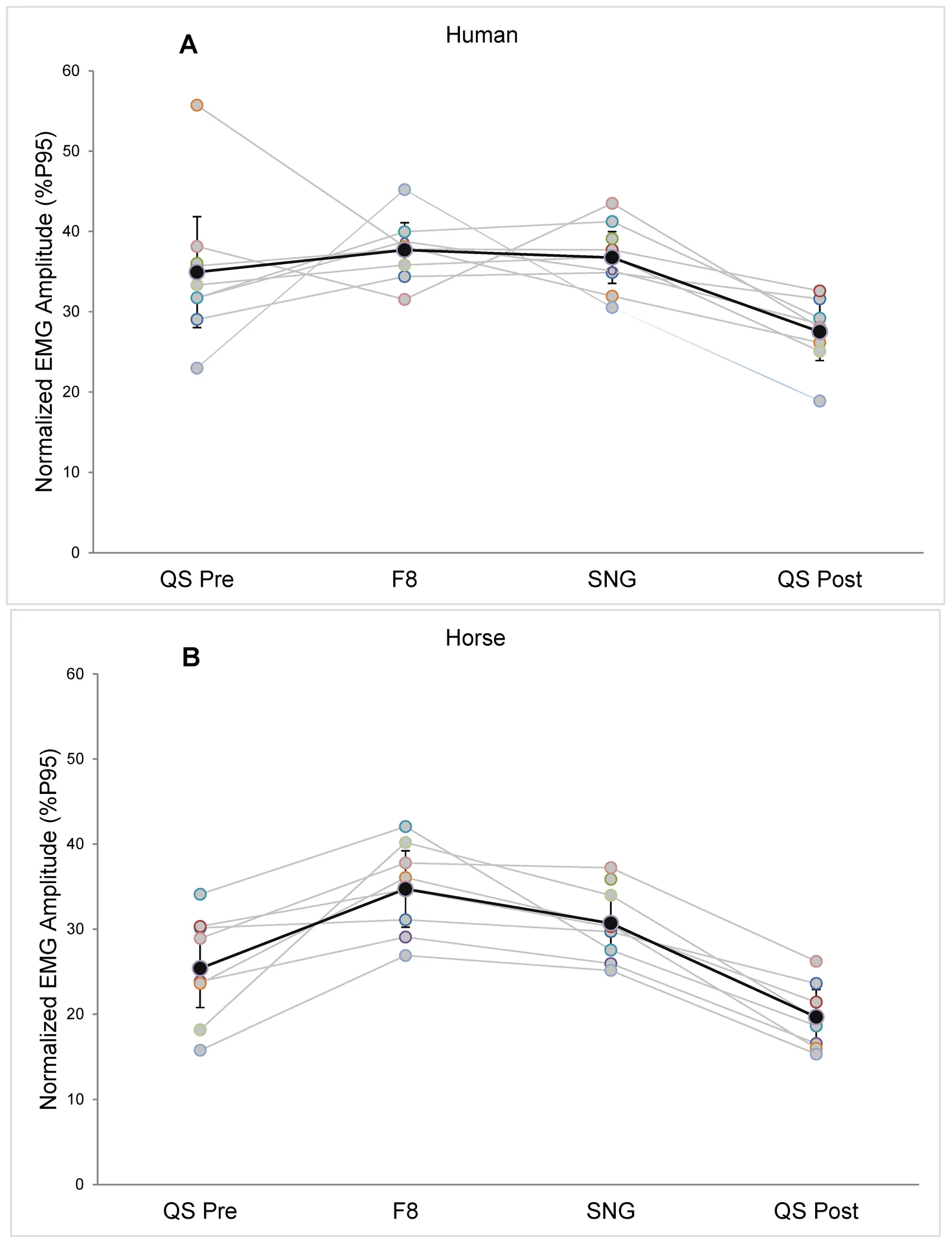
Participant-level normalized EMG amplitude (% of P95) during pre-session quiet sitting (QS Pre), figure-eight riding (F8), stop-and-go riding (SNG), and post-session quiet sitting (QS Post) for humans (**A**) and horses (**B**). Colored markers and connecting lines represent individual participant values averaged across muscles, with consistent colors used to identify the participant across panels A and B. Black markers and lines represent observed condition means, and error bars indicate pointwise 95% confidence intervals. Model-estimated means and contrasts are reported in Table 2. Horse values correspond to equine recordings from each participant’s session.

Across QS Pre, F8, and SNG, human normalized EMG amplitude did not differ significantly by condition, F(2, 63.24) = 0.38, *p* = 0.683. Neither the muscle effect, F(2, 61.61) = 1.12, *p* = 0.331, nor the condition × muscle interaction, F(4, 61.61) = 0.50, *p* = 0.736, was significant. All condition contrasts were nonsignificant (Bonferroni-adjusted *p* = 1.000) (Table 2). Equine normalized EMG amplitude differed by condition, F(2, 85.56) = 10.54, *p* < 0.001. Amplitude was greater during F8 than QS Pre (difference = 9.43 percentage points, 95% CI [4.27, 14.59], Bonferroni-adjusted *p* < 0.001) and during SNG than QS Pre (difference = 5.30 percentage points, 95% CI [0.32, 10.27], Bonferroni-adjusted *p* = 0.033). Within the three-condition model, F8 and SNG did not differ significantly (difference = 4.14 percentage points, 95% CI [−1.02, 9.30], Bonferroni-adjusted *p* = 0.160). Neither the muscle effect, F(3, 84.29) = 2.03, *p* = 0.116, nor the condition × muscle interaction, F(6, 84.29) = 0.33, *p* = 0.921, was significant. In participant-level sensitivity analyses, the condition effect remained nonsignificant for human EMG, F(2, 16.07) = 0.47, *p* = 0.635, and significant for equine EMG, F(2, 15.51) = 13.42, *p* < 0.001. Equine amplitude remained greater during F8 than QS Pre (difference = 9.43 percentage points, 95% CI [3.75, 15.11], Bonferroni-adjusted *p* = 0.001). The SNG–QS Pre difference was similar in magnitude to that in the primary analysis but was not statistically significant (difference = 5.30 percentage points, 95% CI [−0.19, 10.78], Bonferroni-adjusted *p* = 0.060). Individual participant values and observed condition means with pointwise 95% confidence intervals are shown in Figure 2.

### 3.4. Zero-Lag Human–Horse EMG Correlations

***Quiet Sitting Before and After the Session***. Fisher’s z-transformed zero-lag human–horse EMG correlations did not differ between QS Pre and QS Post, F(1, 288.44) = 0.45, *p* = 0.503. Estimated marginal means were 0.0158 for QS Pre and 0.0118 for QS Post (QS Pre—QS Post = 0.0041, 95% CI [−0.0078, 0.0159]) (Table 3).

***Comparison Between Riding Tasks***. In the separate two-condition analysis, zero-lag human–horse EMG correlations differed between F8 and SNG, F(1, 342.92) = 4.50, *p* = 0.035. Estimated marginal means were 0.0011 for F8 and 0.0067 for SNG (F8—SNG = −0.0056, 95% CI [−0.0107, −0.0004], pairwise *p* = 0.034) (Table 3).

***Riding Tasks Compared with Pre-Session Quiet Sitting***. Across QS Pre, F8, and SNG, zero-lag human–horse EMG correlation differed significantly by condition, F(2, 343.50) = 3.36, *p* = 0.036. Estimated marginal means were 0.0147 for QS Pre, 0.0013 for F8, and 0.0067 for SNG. However, none of the Bonferroni-adjusted pairwise comparisons was significant: QS Pre versus F8 (difference = 0.0134, 95% CI [−0.0009, 0.0278], adjusted *p* = 0.074), QS Pre versus SNG (difference = 0.0080, 95% CI [−0.0053, 0.0213], adjusted *p* = 0.442), or F8 versus SNG (difference = −0.0054, 95% CI [−0.0117, 0.0009], adjusted *p* = 0.122) (Table 3). Across conditions, estimated Fisher’s z values were small and close to zero.

Residual diagnostics indicated upper tail departures from normality. Sensitivity analyses excluding the largest positive residual from each model did not alter the inferential conclusions. The QS Pre–QS Post comparison remained nonsignificant, F(1, 309.58) = 0.49, *p* = 0.486; the F8–SNG condition effect remained significant, F(1, 355.56) = 4.47, *p* = 0.035; and the overall condition effect across QS Pre, F8, and SNG remained significant, F(2, 355.17) = 3.07, *p* = 0.048, with no Bonferroni-adjusted pairwise comparison reaching significance.

### 3.5. Lagged Human–Horse EMG Correlations

***Quiet Sitting Before and After the Session***. Absolute lagged human–horse EMG correlation strength within the prespecified ±300 ms window did not differ significantly between QS Pre and QS Post, F(1, 300.65) = 2.55, *p* = 0.111. Estimated marginal means were 0.124 for QS Pre and 0.116 for QS Post (QS Pre—QS Post = 0.008, 95% CI [−0.002, 0.018], pairwise *p* = 0.109) (Table 4).

***Comparison Between Riding Tasks***. Lagged correlation strength did not differ significantly between F8 and SNG, F(1, 348.32) = 2.19, *p* = 0.140. Estimated marginal means were 0.137 for F8 and 0.135 for SNG (F8—SNG = 0.002, 95% CI [−0.001, 0.006], pairwise *p* = 0.139) (Table 4).

***Riding Tasks Compared with Pre-Session Quiet Sitting***. Across QS Pre, F8, and SNG, lagged correlation strength differed significantly by condition, F(2, 342.10) = 4.65, *p* = 0.010. Estimated marginal means were 0.124 for QS Pre, 0.137 for F8, and 0.135 for SNG. Bonferroni-adjusted pairwise comparisons indicated greater lagged correlation strength during F8 than QS Pre (difference = 0.013, 95% CI [0.003, 0.024], adjusted *p* = 0.008) and during SNG than QS Pre (difference = 0.011, 95% CI [0.001, 0.021], adjusted *p* = 0.032). F8 and SNG did not differ significantly (difference = 0.003, 95% CI [−0.001, 0.007], adjusted *p* = 0.374) (Table 4). Residual diagnostics for the three-condition model indicated upper tail departures from normality and several relatively large positive residuals. Excluding the largest positive residual did not alter the interpretation. The condition effect remained significant, F(2, 353.70) = 6.11, *p* = 0.002; F8 remained greater than QS Pre (difference = 0.015, 95% CI [0.005, 0.025], adjusted *p* = 0.002), as did SNG (difference = 0.012, 95% CI [0.003, 0.022], adjusted *p* = 0.007), whereas F8 and SNG did not differ significantly (difference = 0.003, 95% CI [−0.001, 0.007], adjusted *p* = 0.378). Expanding the lag-search window from ±300 to ±500 ms produced the same pattern, with a significant condition effect, F(2, 336.95) = 6.58, *p* = 0.002. F8 remained greater than QS Pre (difference = 0.016, 95% CI [0.006, 0.027], adjusted *p* < 0.001), as did SNG (difference = 0.014, 95% CI [0.004, 0.024], adjusted *p* = 0.003), while F8 and SNG did not differ significantly (difference = 0.003, 95% CI [−0.002, 0.007], adjusted *p* = 0.409).

### 3.6. EMG-EMG Coherence

***Quiet Sitting Before and After the Session***. Median human–horse EMG coherence did not differ significantly overall between QS Pre and QS Post, F(1, 357.56) = 1.00, *p* = 0.318 (Table 5). There was a significant effect of frequency band, F(3, 378.38) = 1281.25, *p* < 0.001, and a significant condition × frequency band interaction, F(3, 353.24) = 4.83, *p* = 0.003. Follow-up comparisons indicated a small increase in alpha coherence from QS Pre to QS Post (difference = 0.00407, Bonferroni-adjusted 95% CI [0.00012, 0.00802], Bonferroni-adjusted *p* = 0.040). Contrast confidence intervals and *p*-values were adjusted across the four frequency bands. No other band-specific comparison remained significant after correction. However, in a sensitivity model allowing participant-specific band effects and Pre–Post changes within bands, the condition effect, F(1, 33.91) = 0.31, *p* = 0.581, and condition × frequency-band interaction, F(3, 22.76) = 1.10, *p* = 0.369, were not significant, although the frequency-band effect remained significant, F(3, 22.15) = 53.63, *p* < 0.001. The alpha difference was also nonsignificant in the sensitivity model (Bonferroni-adjusted *p* = 0.628), indicating that evidence for the small alpha increase was sensitive to model specification.

***Comparison Between Riding Tasks***. Median human–horse EMG coherence differed significantly between F8 and SNG, F(1, 354.90) = 49.99, *p* < 0.001, and across frequency bands, F(3, 382.85) = 2811.98, *p* < 0.001 (Table 5). The condition × frequency band interaction was also significant, F(3, 352.40) = 10.35, *p* < 0.001. Estimated marginal mean coherence was 0.347 for F8 (95% CI [0.337, 0.358]) and 0.350 for SNG (95% CI [0.340, 0.361]). Band-specific comparisons showed greater coherence during SNG than F8 in alpha (difference = 0.00384, Bonferroni-adjusted 95% CI [0.00221, 0.00547]), beta (difference = 0.00457, Bonferroni-adjusted 95% CI [0.00312, 0.00602]), and low gamma (difference = 0.00339, Bonferroni-adjusted 95% CI [0.00216, 0.00462]). All remained significant after Bonferroni correction across the four bands (adjusted *p* < 0.001). High-gamma coherence did not differ between conditions (adjusted *p* = 1.000). In the expanded random effects sensitivity model, the overall condition effect remained significant, F(1, 32.34) = 15.35, *p* < 0.001, as did the frequency band effect, F(3, 19.50) = 116.32, *p* < 0.001, whereas the condition × frequency band interaction was no longer significant, F(3, 23.16) = 2.50, *p* = 0.084. After correction across the four bands, the sensitivity contrasts remained significant for alpha (adjusted *p* = 0.047) and beta (adjusted *p* = 0.008), but not low gamma (adjusted *p* = 0.065) or high gamma (adjusted *p* = 1.000). Thus, the overall SNG–F8 difference was supported by the expanded random effects sensitivity model, whereas evidence that the condition difference varied across frequency bands was sensitive to model specification.

***Riding Tasks Compared with Pre-Session Quiet Sitting***. Median human–horse EMG coherence differed significantly by condition, F(2, 360.31) = 125.62, *p* < 0.001; frequency band, F(3, 384.09) = 2499.00, *p* < 0.001; and their interaction, F(6, 297.57) = 16.58, *p* < 0.001 (Table 5). Estimated marginal mean coherence was 0.364 for QS Pre, 0.348 for F8, and 0.351 for SNG. Bonferroni-adjusted comparisons indicated greater coherence during QS Pre than F8 (difference = 0.016, 95% CI [0.013, 0.018], *p* < 0.001) and SNG (difference = 0.013, 95% CI [0.011, 0.015], *p* < 0.001), and greater coherence during SNG than F8 (difference = 0.003, 95% CI [0.002, 0.004], *p* < 0.001). These overall contrast confidence intervals and *p*-values were adjusted for the three unique condition comparisons. Band-specific comparisons showed greater QS Pre coherence than both riding conditions across all four frequency bands. SNG also exceeded F8 in alpha, beta, and low-gamma coherence (all adjusted *p* < 0.001), whereas high-gamma coherence did not differ between F8 and SNG (SNG—F8 difference = −0.00021, Bonferroni-adjusted 95% CI [−0.00201, 0.00160], adjusted *p* = 1.000). For these band-specific contrasts, Bonferroni correction covered the three unique condition comparisons within each band, without additional correction across bands.

## 4. Discussion

The present study provides, to our knowledge, the first simultaneous characterization of human and equine trunk muscle activity during PT utilizing equine movement in individuals with PD using surface EMG. Four complementary features of the recorded signals were examined: normalized EMG amplitude, simultaneous human–horse temporal covariation, lagged human–horse temporal covariation, and frequency-specific human–horse covariation. Human normalized EMG amplitude remained relatively stable across riding conditions, whereas equine amplitude differed more clearly across tasks. Zero-lag human–horse correlations were generally very small, despite a modest difference between the riding tasks in the direct comparison. When short temporal offsets were permitted, both riding conditions showed greater human–horse covariation than pre-session quiet sitting. EMG-EMG coherence showed a different pattern, with greater coherence during pre-session quiet sitting than during riding and greater coherence during SNG than F8 riding overall. Together, these findings indicate that riding conditions were associated with differences in several distinct properties of human and equine EMG signals and their cross-species covariation, whereas overall human trunk EMG magnitude changed comparatively little. These differences should be interpreted descriptively because the study cannot distinguish neuromuscular adaptation from mechanical and task-related influences such as locomotion, turning, stopping, handler guidance, or session order.

### 4.1. Human and Equine EMG Amplitude Across Sequential Conditions

Human and equine EMG amplitude showed different patterns across conditions (Table 2; Figure 2). In participants with PD, normalized trunk EMG amplitude did not differ between F8 and SNG riding, nor did either riding condition differ from pre-session QS. Human amplitude also decreased from pre- to post-session QS, although this difference did not reach statistical significance. Thus, within the sampled trunk muscles, the riding conditions were not associated with a clear increase in overall human EMG amplitude. This does not indicate that the riding tasks imposed no postural or sensorimotor demands. Horseback riding requires ongoing postural regulation while seated on a moving base of support, and rider postural behavior can vary with task and sensory conditions [57]. Rather, normalized EMG amplitude may not capture changes in the timing or organization of muscle activity that occur during this postural regulation.

The equine amplitude findings showed greater differences across conditions. Equine amplitude was greater during both riding conditions than during QS Pre in the three-condition model, although the SNG-QS Pre difference was not statistically significant in the participant-level sensitivity analysis. F8 also exceeded SNG in the direct two-condition comparison, but this difference was not statistically significant in the adjusted three-condition model. Equine amplitude was also lower during post-session than pre-session QS. These observed differences may reflect changes in the mechanical demands experienced by the horse across stationary and mounted locomotion conditions [18,19]. Continuous locomotion, turning, repeated stopping and restarting, handler guidance, and changes in gait characteristics may all contribute to differences in recorded muscle activity. Because the SNG analysis windows incorporated stopping and restarting, whereas F8 windows involved continuous walking, differences in the proportion of time spent moving may have contributed to the observed equine amplitude differences. Additionally, walking speed and cadence were not experimentally standardized, and F8 riding always preceded SNG riding. Consequently, the present data cannot determine whether the observed amplitude differences between riding conditions reflected specific task characteristics, changes in locomotor behavior, or time-dependent effects across the session. Furthermore, SNG incorporated ring handling and verbal commands in addition to stopping and restarting, preventing the contributions of these.

Previous studies have characterized trunk and lower limb muscle activation during EAS interventions in different populations. Differences in trunk muscle activation have been reported in older adults [29], while changes in trunk and lower-limb EMG activity have been observed across gait and hippotherapy conditions in children with cerebral palsy [28]. Additionally, EMG activity has been examined during human gait and hippotherapy using different riding materials in healthy adults [58]. Although these populations and protocols differ from the present study, they support the broader observation that muscle activation during EAS can vary across experimental and clinical contexts. The present findings extend this work by showing that simultaneously recorded human and horse trunk EMG did not change in parallel across conditions. Human mean normalized amplitude remained comparatively stable, whereas equine amplitude showed clearer differences between stationary and riding conditions, with less consistent statistical evidence for differences between the sequential riding conditions (Table 2; Figure 2).

The lower equine amplitude during QS Post compared with QS Pre should also be interpreted cautiously. Because the quiet-sitting measurements occurred at different points in the session, any pre-to-post difference could reflect time-dependent influences, including habituation, fatigue, changes in posture, or other session-related factors, rather than a specific effect of the intervening riding activity [59,60]. Similarly, the near-significant reduction in human amplitude from QS Pre to QS Post does not provide sufficient evidence for a PT-related change. These QS Pre to QS Post observations are therefore best considered descriptive within this single-session exploratory design.

### 4.2. Human–Horse Temporal Covariation

Zero-lag human–horse correlations were generally very small, indicating little simultaneous linear covariation between the recorded human and equine EMG signals (Table 3). Although SNG riding showed slightly greater positive zero-lag covariation than F8 riding in the direct comparison, the absolute effect was small. In the three-condition analysis (QS Pre, F8, SNG), the overall condition effect was significant, but no pairwise comparison remained significant after adjustment. These findings therefore provide limited evidence for meaningful task-related differences in strictly simultaneous human–horse EMG covariation.

A different pattern emerged when temporal offsets were permitted. Lagged correlation strength within the prespecified ±300 ms window was greater during both riding conditions than during QS Pre, whereas F8 and SNG did not differ from one another (Table 4). These findings were preserved when the lag window was expanded to ±500 ms and after removal of the largest positive residual. Together, these results indicate greater values of the selected lagged-correlation measure during riding than during QS Pre, although the extent to which these differences reflect meaningful cross-signal coupling remains uncertain. This is plausible because related patterns of muscle activity and mechanically mediated postural responses need not occur simultaneously [38,39,40,51]. During mounted activity, locomotion, turning, stopping, handler guidance, and rider postural adjustments could all contribute to such temporal offsets. However, because lagged correlation was defined as the maximum absolute correlation within a prespecified window, nonzero values may arise even in the absence of meaningful cross-signal coupling. The observed between-condition differences were not evaluated against surrogate-derived null distributions, and differences in the temporal and spectral characteristics of the EMG signals may have influenced these estimates. Furthermore, lagged cross-correlation cannot identify the source, direction, or physiological mechanism of these associations. The observed differences should therefore be interpreted as temporal covariation across sequentially administered conditions rather than evidence that one member of the dyad led, drove, or entrained the other.

Previous studies of human–horse interaction have shown that coordination can vary with riding condition when assessed using kinematic measures [24,25,26,27]. The present findings extend this work by demonstrating that simultaneously recorded human–horse EMG signals also exhibit differences in temporal covariation across sequentially administered conditions. However, the very small zero-lag correlations and the exploratory single-session design warrant caution in assigning biological significance to these effects. Future studies combining simultaneous EMG with synchronized kinematics and controlled measures of gait speed and cadence will be needed to determine how much of the observed temporal covariation reflects shared mechanical task structure versus more specific neuromuscular interactions.

### 4.3. Frequency-Specific Human–Horse Covariation

**Figure 3:**
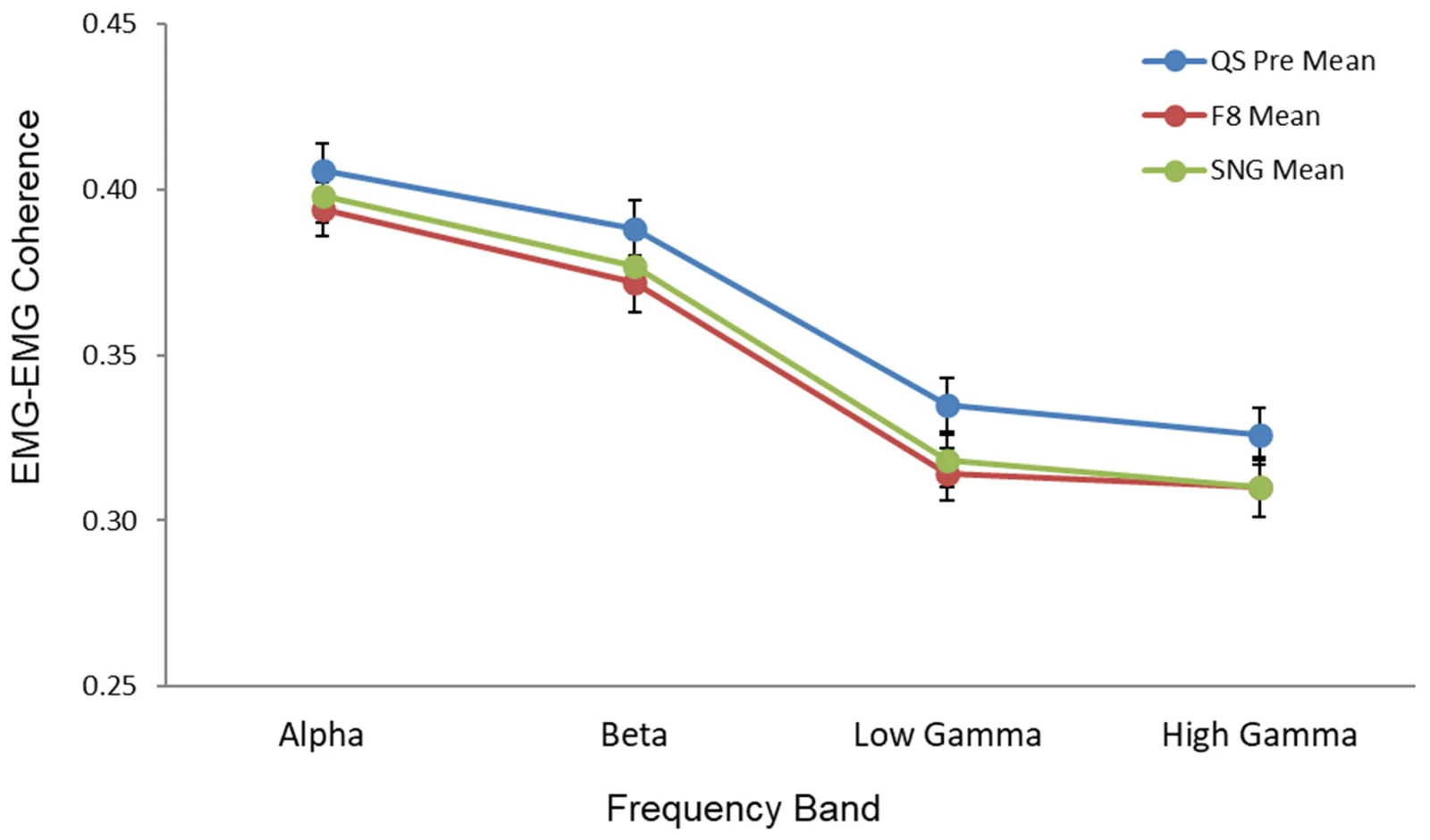
Human–horse EMG coherence across frequency bands during pre-session quiet sitting (QS Pre), figure-eight riding (F8), and stop-and-go riding (SNG). Coherence was derived from the three-condition mixed model. Error bars represent pointwise 95% confidence intervals around the estimated marginal means.

EMG-EMG coherence revealed a different pattern from the time-domain correlation analyses (Figure 3, Table 5). In the three-condition comparison, coherence was greater during QS Pre than during either riding condition across all four frequency bands. Within the riding conditions, SNG riding showed greater coherence than F8 riding in the alpha, beta, and low-gamma ranges, whereas no difference was observed in the high-gamma ranges. However, the Condition × Frequency band interaction was attenuated in the expanded random effects sensitivity model. These findings suggest differences in human–horse EMG coherence across the sequential experimental conditions. However, the sensitivity of some effects to model specification, together with the limited sample size and inability to obtain a converged expanded three-condition sensitivity model, warrants caution regarding the consistency and generalizability of the observed frequency-specific patterns.

The higher coherence observed during QS Pre should not be interpreted as evidence of stronger or more effective human–horse coordination. EMG-EMG coherence reflects the consistency of shared frequency-specific signal structure, whereas lagged cross-correlation quantifies temporal covariation while allowing for offsets between signals. These measures therefore capture different properties of the EMG signals and need not vary in the same direction. The greater lagged correlation during riding, together with lower coherence during riding than QS Pre, may indicate that mounted activity introduced more temporally variable or broadband relationships between human and equine muscle activity rather than a stable frequency-specific association. This interpretation remains tentative because the present study did not directly test the mechanical or physiological sources of the observed signal relationships.

Observed differences in coherence may also reflect changes in the mechanical demands and movement structure of the activity [18,19]. During QS, both members of the dyad were relatively stationary, whereas riding introduced locomotion, turning, repeated postural adjustments, and, during SNG, stopping and restarting. These task features could alter the periodicity and spectral content of the recorded EMG signals, potentially influencing coherence estimates independently of any specific physiological interaction between horse and rider [61]. Because horse speed and cadence were not experimentally standardized, their contribution to the observed coherence patterns cannot be separated from other condition-related or time-dependent effects.

The frequency band findings require additional caution. Alpha, beta, low-gamma, and high-gamma bands were applied identically to human and equine EMG as an exploratory framework, but corresponding physiological frequency band classifications have not been established for equine EMG. Accordingly, the band labels should be interpreted descriptively rather than as evidence of equivalent neural or physiological mechanisms across species. Future studies combining simultaneous EMG with controlled gait speed, cadence, and synchronized kinematic measures will be needed to determine whether observed differences in coherence across conditions reflect shared mechanical rhythms, neuromuscular interactions, or a combination of both.

### 4.4. Interpretation and Implications of Differences Across Sequential Conditions

Taken together, the findings indicate that human and equine EMG activity differed across the sequentially administered conditions, but these differences were not expressed uniformly across outcomes. Human normalized EMG amplitude changed little across riding conditions, whereas equine amplitude, lagged human–horse covariation, and frequency-specific covariation exhibited differences across conditions, although the statistical support for some comparisons varied across models. This pattern reinforces that signal magnitude, simultaneous temporal covariation, lagged temporal covariation, and coherence characterize different features of neuromuscular behavior and should not be expected to change in parallel.

The findings can be considered within a constraints-based framework in which observed behavior reflects the interaction of characteristics of the individual, task, and environment [23]. In the present study, F8 and SNG differed in several task features, including continuous versus interrupted locomotion, repeated stopping and restarting, verbal commands, and holding a rubber ring during SNG. These differences were embedded within a fixed task sequence and occurred within a broader mechanical context that included horse gait, turning, handler guidance, and rider postural responses. Accordingly, the observed EMG differences may reflect the combined influences of task characteristics, mechanical conditions, and time-dependent factors, but the present design cannot determine their individual contributions or establish whether they represent adaptive neuromuscular regulation. Coordination dynamics therefore provides a descriptive framework for considering the results rather than a mechanism directly tested in this study.

These findings may nevertheless help generate hypotheses for future rehabilitation research. Individuals with PD commonly experience impairments in postural control and adaptability [11,55,62,63], making the neuromuscular demands associated with different mounted activities potentially relevant to treatment design. However, the present single-session study did not assess whether the observed EMG patterns were beneficial, whether they contributed to clinical improvement, or whether modifying mounted task constraints can systematically alter therapeutic demand. Future studies should test these possibilities directly using counterbalanced task designs, controlled gait characteristics, larger samples of riders and horses, and concurrent biomechanical and clinical outcome measures.

### 4.5. Limitations and Future Directions

Several limitations should be considered when interpreting these findings. First, this exploratory study included nine participants with PD and three therapy horses, with the same horses used across sessions. This limits statistical power, constrains generalization to the broader PD population and to other therapy horses, and prevents horse-specific effects from being characterized reliably. The absence of a healthy control group also means that the observed EMG patterns cannot be considered specific to PD.

Second, F8 riding always preceded SNG riding. Task and order were therefore confounded, and differences between the riding conditions cannot be separated from potential effects of fatigue, habituation, medication timing, or other time-dependent changes across the session [59,60,64]. Furthermore, SNG included additional components, such as holding a rubber ring, responding to verbal commands, and repeated stopping and restarting, whose individual contributions cannot be isolated. In addition, horse walking speed and cadence were not experimentally standardized, and participant body mass, although restricted by eligibility criteria, was not modeled as a continuous measure of rider load. Differences in gait characteristics, turning, stopping, handler guidance, and rider load may therefore have contributed to the observed differences in EMG activity across conditions. Future studies should counterbalance task order and directly quantify or control gait speed, cadence, and rider load.

Third, this study characterized a single treatment session and was not designed as a clinical effectiveness trial. The observed differences in EMG amplitude, temporal covariation, and coherence cannot establish whether these patterns are therapeutically beneficial, whether they change with repeated treatment, or whether they are related to improvements in balance, gait, or other clinical outcomes. Longitudinal studies combining simultaneous human–horse EMG with clinical outcomes are needed to determine the functional relevance of these measures.

Several measurement limitations should also be acknowledged. Surface EMG is restricted to superficial musculature and is susceptible to cross-talk [61,65], and only selected trunk muscles were recorded in the human and horse. A dedicated ECG-removal procedure was not applied, so residual cardiac contamination of trunk EMG cannot be completely excluded [66,67]. In addition, the alpha, beta, low-gamma, and high-gamma bands used for coherence were derived from frequency ranges previously applied in human EMG coherence research [41,54,55]. Because corresponding physiological band classifications have not been established for equine EMG, these bands should be interpreted as an exploratory descriptive framework rather than as evidence of equivalent physiological mechanisms across species.

Finally, correlation and coherence quantify statistical relationships between signals but do not establish causal direction, shared neural drive, or direct physiological coupling. The very small zero-lag correlations in the present study warrant particular caution in assigning biological importance to statistically significant comparisons. Future work should combine simultaneous EMG with synchronized three-dimensional kinematics, gait timing, and kinetic measures to determine how much of the observed cross-species covariation reflects shared mechanical structure versus more specific neuromuscular interactions.

## 5. Conclusions

Simultaneous human and equine surface EMG was feasible during physical therapy utilizing equine movement and revealed differences in EMG amplitude and human–horse temporal and frequency-specific covariation across sequentially administered conditions. These measures showed different patterns across conditions, although the contributions of task characteristics and order-related influences could not be distinguished. These exploratory single-session findings require confirmation in larger studies using counterbalanced task orders, greater numbers of horses and participants, controlled gait characteristics, and relevant clinical outcomes.

## Figures and Tables

**Table 1:** Descriptive characteristics of study participants and horses. Data are presented as mean ± standard deviation (SD) or number (*n*), as appropriate. Parkinson’s disease duration was calculated from the date of diagnosis to the baseline assessment. MMSE = Mini-Mental State Examination; MDS-UPDRS Part III = Movement Disorder Society Unified Parkinson’s Disease Rating Scale Part III (Motor Examination).

Characteristic	Participants (*n* = 9)	Horses (*n* = 3)
Age, years	73.9 ± 5.7	17.3 ± 3.2
Sex, ***n***	Male: 5; Female: 4	Gelding: 2; Mare: 1
Breed	—	Quarter Horse
Parkinson’s disease duration, years	5.7 ± 3.7	—
Hoehn and Yahr stage	2.9 ± 0.5	—
MDS-UPDRS Part III	28.1 ± 20.4	—
MMSE	28.2 ± 2.2	—

**Table 2:** Normalized human and equine EMG amplitude across experimental conditions. Estimated marginal means are expressed as percentages of the channel-specific 95th percentile reference (P95); between-condition differences are expressed in percentage points. Mean confidence intervals are pointwise 95% intervals. Contrast confidence intervals and *p*-values use Bonferroni adjustment within each model and species, covering one unique comparison in each two-condition model and three in each three-condition model. Estimated marginal means are percentages of P95. Differences are percentage points. Values are reported separately for each fitted model.

**Quiet Sitting Before and After the Session**		
**Estimate**	**Human [95% CI]**	**Equine [95% CI]**
QS Pre	34.94 [29.81, 40.06]	25.39 [21.52, 29.25]
QS Post	27.50 [22.02, 32.99]	19.61 [15.51, 23.71]
Post—Pre; pairwise *p*	−7.43 [−15.02, 0.15]; 0.055	−5.77 [−10.70, −0.85]; 0.022
**Comparison Between Riding Tasks**		
**Estimate**	**Human [95% CI]**	**Equine [95% CI]**
F8	37.57 [35.17, 39.98]	35.11 [31.56, 38.66]
SNG	36.76 [34.50, 39.02]	30.68 [27.23, 34.14]
F8—SNG; pairwise *p*	0.81 [−3.27, 4.89]; 0.690	4.43 [1.44, 7.41]; 0.004
**Riding Tasks Compared with Pre-Session Quiet Sitting**		
**Estimate**	**Human [95% CI]**	**Equine [95% CI]**
QS Pre	34.94 [30.92, 38.96]	25.39 [21.84, 28.94]
F8	37.61 [33.32, 41.90]	34.82 [31.10, 38.55]
SNG	36.76 [32.74, 40.78]	30.68 [27.13, 34.23]
F8—QS Pre; adjusted *p*	2.67 [−5.29, 10.64]; 1.000	9.43 [4.27, 14.59]; <0.001
SNG—QS Pre; adjusted *p*	1.82 [−5.96, 9.60]; 1.000	5.30 [0.32, 10.27]; 0.033
F8—SNG; adjusted *p*	0.85 [−7.11, 8.81]; 1.000	4.14 [−1.02, 9.30]; 0.160

**Note.** Mean confidence intervals are pointwise. Pairwise *p*-values and contrast confidence intervals use the SPSS Bonferroni adjustment within each model and species: one unique comparison for each two-condition model and three for each three-condition model. P95 = 95th percentile; QS = quiet sitting; F8 = figure-8 riding; SNG = stop-and-go riding.

**Table 3:** Zero-lag human–horse EMG correlations across experimental conditions. Estimated marginal means and between-condition differences are reported on the Fisher’s z scale for signed Pearson correlations between paired human and equine EMG linear envelopes. Mean confidence intervals are pointwise 95% intervals. Contrast confidence intervals and *p*-values use Bonferroni adjustment within each model. Model summaries report condition effects and participant and muscle-pair random intercept variances.

**Analysis**	**Condition**	**Estimated Marginal Mean, Fisher’s z (95% CI)**	**Contrast**	**Difference**	**95% CI**	**Adj. *p***
Quiet sitting before vs. after session	QS Pre	0.0158 [−0.0140, 0.0457]	QS Pre—QS Post	0.0041	[−0.0078, 0.0159]	0.501
	QS Post	0.0118 [−0.0181, 0.0416]				
Comparison between riding tasks	F8	0.0011 [−0.015, 0.017]	F8—SNG	−0.0056	[−0.0107, −0.0004]	0.034
	SNG	0.0067 [−0.009, 0.023]				
Riding tasks compared with pre-session quiet sitting	QS Pre	0.0147 [−0.0032, 0.0326]	QS Pre—F8	0.0134	[−0.0009, 0.0278]	0.074
	F8	0.0013 [−0.0155, 0.0181]	QS Pre—SNG	0.0080	[−0.0053, 0.0213]	0.442
	SNG	0.0067 [−0.0100, 0.0234]	F8—SNG	−0.0054	[−0.0117, 0.0009]	0.122
**Model summary**						
**Analysis**	**Condition Effect**		**Participant Random** **Intercept Variance**		**Muscle Pair Random** **Intercept Variance**	
Quiet sitting before vs. after session	F(1, 288.45) = 0.45, *p* = 0.503		0.0012875		0.0000493	
Comparison between riding tasks	F(1, 342.92) = 4.50, *p* = 0.035		0.0003342		0.0003946	
Riding tasks compared with pre-session quiet sitting	F(2, 343.50) = 3.36, *p* = 0.036		0.0003835		0.0002183	

**Note.** Values are Fisher’s z-transformed zero-lag Pearson correlations between paired human and horse EMG linear envelopes. Estimated marginal means and contrasts are reported separately for each fitted model. Pairwise comparisons are Bonferroni-adjusted. Participant and Muscle Pair values are random intercept variance estimates. QS Pre = pre-session quiet sitting; QS Post = post-session quiet sitting; F8 = figure-eight riding; SNG = stop-and-go riding.

**Table 4:** Lagged human–horse EMG correlations across experimental conditions. Estimated marginal means and between-condition differences describe absolute correlation strength within the ±300 ms lag-search window and are reported without Fisher’s z transformation. Mean confidence intervals are pointwise 95% intervals. Contrast confidence intervals and *p*-values use Bonferroni adjustment within each model. Model summaries report condition effects and participant and muscle-pair random intercept variances.

**Analysis**	**Condition**	**Estimated Marginal Mean (95% CI)**	**Contrast**	**Difference (95% CI)**	**Adjusted *p***
Quiet sitting before vs. after session	QS Pre	0.124 [0.104, 0.145]	QS Pre—QS Post	0.008 [−0.002, 0.018]	0.109
	QS Post	0.116 [0.096, 0.137]			
Comparison between riding tasks	F8	0.137 [0.127, 0.147]	F8—SNG	0.002 [−0.001, 0.006]	0.139
	SNG	0.135 [0.125, 0.144]			
Riding tasks compared with pre-session quiet sitting	QS Pre	0.124 [0.111, 0.137]	QS Pre—F8	−0.013 [−0.024, −0.003]	0.008
	F8	0.137 [0.126, 0.149]	QS Pre—SNG	−0.011 [−0.021, −0.001]	0.032
	SNG	0.135 [0.124, 0.146]	F8—SNG	0.003 [−0.001, 0.007]	0.374
**Model summary**					
**Analysis**		**Condition effect**		**Participant variance**	**Muscle pair variance**
Quiet sitting before vs. after session		F(1, 300.65) = 2.55, *p* = 0.111		0.0005502	0.00003768
Comparison between riding tasks		F(1, 348.32) = 2.19, *p* = 0.140		0.000105	0.000118
Riding tasks compared with pre-session quiet sitting		F(2, 342.10) = 4.65, *p* = 0.010		0.000169	0.000101

**Note.** Primary lagged-correlation analyses used the prespecified ±300 ms lag window. Estimated marginal means and contrasts are reported separately for each fitted model. Pairwise comparisons are Bonferroni-adjusted. Participant and muscle pair values are random intercept variance estimates. QS Pre = pre-session quiet sitting; QS Post = post-session quiet sitting; F8 = figure-eight riding; SNG = stop-and-go riding. Sensitivity analyses excluding the largest positive residual and expanding the lag-search window to ±500 ms are reported in the manuscript.

**Table 5:** Human–horse EMG-EMG coherence across experimental conditions and frequency bands. Estimates describe the mean of trial-level median wavelet coherence. The table presents overall and band-specific estimated marginal means (EMMs), between-condition contrasts, fixed effects, random intercept variances, and expanded random effects sensitivity results. Mean confidence intervals are pointwise 95% intervals. Two-condition band-specific contrasts use Bonferroni adjustment across four bands; three-condition band-specific contrasts use Bonferroni adjustment across three unique condition comparisons within each band, without additional adjustment across bands. Overall contrasts use one comparison in each two-condition model and Bonferroni adjustment across three comparisons in the three-condition model. Estimated marginal means and contrasts are reported separately for each fitted model. All analyses are exploratory.

**Overall Estimated Marginal Means**						
**Analysis**		**Condition**	**EMM Coherence**		**Pointwise 95% CI**	
QS Pre vs. QS Post		QS Pre	0.36337		[0.35526, 0.37147]	
QS Pre vs. QS Post		QS Post	0.36414		[0.35606, 0.37222]	
F8 vs. SNG		F8	0.34748		[0.33728, 0.35768]	
F8 vs. SNG		SNG	0.35037		[0.34020, 0.36055]	
QS Pre vs. F8 vs. SNG		QS Pre	0.36358		[0.35555, 0.37162]	
QS Pre vs. F8 vs. SNG		F8	0.34773		[0.33967, 0.35579]	
QS Pre vs. F8 vs. SNG		SNG	0.35060		[0.34257, 0.35862]	
**Overall condition contrasts**						
**Analysis**		**Contrast**	**Difference**		**95% CI**	***p***
QS Pre vs. QS Post		QS Post— QS Pre	0.00078		[−0.00074, 0.00229]	0.316
F8 vs. SNG		SNG—F8	0.00290		[0.00209, 0.00370]	<0.001
QS Pre vs. F8 vs. SNG		QS Pre—F8	0.01586		[0.01345, 0.01826]	<0.001
QS Pre vs. F8 vs. SNG		QS Pre—SNG	0.01299		[0.01092, 0.01506]	<0.001
QS Pre vs. F8 vs. SNG		SNG—F8	0.00287		[0.00189, 0.00384]	<0.001
**Fixed effects and random intercept variances**						
**Analysis**	**Condition**	**Frequency Band**	**Condition × Band**		**Participant Variance**	**Muscle Pair Variance**
QS Pre vs. QS Post	F(1, 357.56) = 1.00 *p* = 0.318	F(3, 378.38) = 1281.25 *p* < 0.001	F(3, 353.24) = 4.83 *p* = 0.003		9.65767 × 10^−5^	5.32216 × 10^−5^
F8 vs. SNG	F(1, 354.90) = 49.99 *p* < 0.001	F(3, 382.85) = 2811.98 *p* < 0.001	F(3, 352.40) = 10.35 *p* < 0.001		0.00016404	1.89156 × 10^−5^
QS Pre vs. F8 vs. SNG	F(2, 360.31) = 125.62 *p* < 0.001	F(3, 384.09) = 2499.00 *p* < 0.001	F(6, 297.57) = 16.58 *p* < 0.001		9.78561 × 10^−5^	2.56712 × 10^−5^
**Two-condition band-specific estimates and contrasts**						
**Con-trast**	**Band**	**Reference EMM Pointwise 95% CI**	**Comparison EMM Pointwise 95% CI**	**Difference**	**Bonferroni 95% CI**	**Adj. *p***
QS Post—QS Pre	Alpha	0.40527 [0.39688, 0.41365]	0.40934 [0.40104, 0.41764]	0.00407	[0.00012, 0.00802]	0.040
QS Post—QS Pre	Beta	0.38878 [0.38024, 0.39733]	0.39053 [0.38210, 0.39897]	0.00175	[−0.00147, 0.00497]	0.693
QS Post—QS Pre	Low gamma	0.33467 [0.32661, 0.34273]	0.33295 [0.32491, 0.34099]	−0.00172	[−0.00375, 0.00031]	0.135
QS Post—QS Pre	High gamma	0.32474 [0.31601, 0.33347]	0.32375 [0.31495, 0.33254]	−0.00100	[−0.00322, 0.00122]	1.000
SNG—F8	Alpha	0.39426 [0.38409, 0.40443]	0.39810 [0.38793, 0.40826]	0.00384	[0.00221, 0.00547]	<0.001
SNG—F8	Beta	0.37188 [0.36156, 0.38220]	0.37645 [0.36617, 0.38673]	0.00457	[0.00312, 0.00602]	<0.001
SNG—F8	Low gamma	0.31407 [0.30389, 0.32424]	0.31745 [0.30732, 0.32759]	0.00339	[0.00216, 0.00462]	<0.001
SNG—F8	High gamma	0.30971 [0.29897, 0.32046]	0.30949 [0.29890, 0.32009]	−0.00022	[−0.00213, 0.00169]	1.000
**Expanded random effects sensitivity analyses**						
**Analysis**		**Condition**	**Frequency Band**		**Condition × Band**	
QS Pre vs. QS Post		F(1, 33.91) = 0.31 *p* = 0.581	F(3, 22.15) = 53.63 *p* < 0.001		F(3, 22.76) = 1.10 *p* = 0.369	
F8 vs. SNG		F(1, 32.34) = 15.35 *p* < 0.001	F(3, 19.50) = 116.32 *p* < 0.001		F(3, 23.16) = 2.50 *p* = 0.084	
**Analysis**		**Alpha Adj. *p***	**Beta** **Adj.** ***p***		**Low** **Gamma** **Adj. *p***	**High Gamma Adj. *p***
QS Pre vs. QS Post		0.628	1.000		1.000	1.000
F8 vs. SNG		0.047	0.008		0.065	1.000
**Three-condition band-specific estimated marginal means**						
**Band**		**QS Pre EMM Pointwise 95% CI**	**F8 EMM Pointwise 95% CI**		**SNG EMM Pointwise 95% CI**	
Alpha		0.40586 [0.39754, 0.41418]	0.39445 [0.38643, 0.40247]		0.39832 [0.39030, 0.40635]	
Beta		0.38841 [0.37992, 0.39690]	0.37219 [0.36393, 0.38044]		0.37667 [0.36847, 0.38487]	
Low gamma		0.33455 [0.32656, 0.34254]	0.31435 [0.30633, 0.32238]		0.31768 [0.30970, 0.32565]	
High gamma		0.32552 [0.31687, 0.33416]	0.30992 [0.30112, 0.31873]		0.30972 [0.30110, 0.31833]	
**Three-condition band-specific contrasts**						
**Band**		**Contrast**	**Difference**		**Bonferroni 95% CI**	**Adjusted *p***
Alpha		QS Pre—F8	0.01141		[0.00769, 0.01513]	<0.001
Alpha		QS Pre—SNG	0.00754		[0.00416, 0.01092]	<0.001
Alpha		SNG—F8	0.00387		[0.00231, 0.00543]	<0.001
Beta		QS Pre—F8	0.01623		[0.01265, 0.01980]	<0.001
Beta		QS Pre—SNG	0.01174		[0.00847, 0.01502]	<0.001
Beta		SNG—F8	0.00448		[0.00310, 0.00587]	<0.001
Low gamma		QS Pre—F8	0.02020		[0.01744, 0.02295]	<0.001
Low gamma		QS Pre—SNG	0.01687		[0.01451, 0.01923]	<0.001
Low gamma		SNG—F8	0.00332		[0.00215, 0.00450]	<0.001
High gamma		QS Pre—F8	0.01559		[0.01261, 0.01858]	<0.001
High gamma		QS Pre—SNG	0.01580		[0.01323, 0.01837]	<0.001
High gamma		SNG—F8	−0.00021		[−0.00201, 0.00160]	1.000

**Note.** Differences are reported in the direction indicated by each contrast. QS = quiet sitting; F8 = figure-eight riding; SNG = stop-and-go riding; CI = confidence interval. Frequency bands: alpha (5–13 Hz), beta (13–30 Hz), low gamma (30–60 Hz), and high gamma (60–100 Hz). Coherence is unitless.

## Data Availability

Data supporting the findings of this research are available from the corresponding author.

## Supplementary Materials

The following supporting information can be downloaded at: https://www.mdpi.com/article/10.3390/ani16213328/s1. Supplementary Table S1 Summary of EMG Quality-Control Outcomes, Trial Availability, Notch Filtering, and Valid Model Observations.
