# Supplementary material for: Human–Horse Neuromuscular Coordination Across Sequential Conditions During Physical Therapy Utilizing Equine Movement in Parkinson’s Disease
Source: Animals (Basel). 2026 Oct 22;16(21):3328. doi: 10.3390/ani16213328 (PMC13761152; doi:10.3390/ani16213328)
Supplement: Supplementary file 1 [file animals-16-03328-s001.zip › animals-4599147-supplementary.pdf]

Supplementary Table S1. Summary of EMG Quality-Control Outcomes, Trial Availability, Notch Filtering, and Valid Model Observations

Counts refer to channel-trial records, experimental trials, or model observations as specified and should not be interpreted as independent participants or trials.

Overall quality control summary

QC outcome	n	% of available records	Interpretation
Available channel-trial records	2,296	100.0%	Records present in source data and evaluated by QC
Retained	2,187	95.3%	Passed QC and retained for analysis
Excluded	109	4.7%	Failed QC and excluded from analyses
Clipping	97	4.2%	QC exclusion reason
High-frequency contamination	12	0.5%	QC exclusion reason
Unavailable/not present in source data	350	—	Not evaluated by QC; not counted as QC failures

Quality control summary by condition

Condition	Available	Retained	Excluded	Retention %	Unavailable
QS (Pre + Post)	238	227	11	95.4%	14
F8	1,008	964	44	95.6%	126
SNG	1,050	996	54	94.9%	210
**Overall**	**2,296**	**2,187**	**109**	**95.3%**	**350**

Participant and horse level trial availability

Participant	Horse	QS trials	F8 trials	SNG trials
1	A	2	9	6
2	B	2	9	8
3	C	1	0	7
4	A	2	9	9
5	B	2	9	9
6	C	2	9	10
7	A	2	9	8
8	B	2	9	9
9	C	2	9	9
**Total**	**3 horses**	**17**	**72**	**75**

Note. Trial counts indicate available source trials before channel-specific QC; QS combines pre- and post-session sitting. Participant 3's F8 and QS Post recordings were unavailable following a data-saving failure.

Conditional 60 Hz notch filtering by condition

Condition	Available EMG records	Notch applied (n)	Notch applied (%)
QS Pre	126	21	16.7%
QS Post	112	21	18.8%
F8	1,008	6	0.6%
SNG	1,050	16	1.5%
**Overall**	**2,296**	**64**	**2.8%**

Note. Percentages use available EMG channel trial records before final QC exclusions. Non-EMG S channels were excluded. Conditional notch filtering was applied when the 60 Hz spectral-prominence ratio exceeded 2.0. Differences in filtering prevalence do not establish whether filtering altered coherence comparisons.

Valid observations entering the final mixed effects models

Outcome	Model observation unit	QS Pre	QS Post	F8	SNG
Human normalized EMG amplitude	Participant × condition × muscle	27	24	24	27
Equine normalized EMG amplitude	Participant × condition × muscle	36	32	32	36
Zero-lag human–horse correlation	Participant × condition × side-specific muscle pair	386	356	356	392
Lagged human–horse correlation	Participant × condition × side-specific muscle pair	386	356	356	392
Human–horse EMG coherence	Participant × condition × side-specific muscle pair × frequency band	1,544	1,424	1,424	1,568

Note. Available records were channel trial records present in source data and evaluated by quality control (QC); retention rates use these records as the denominator. Unavailable entries represent trial-by-channel placeholders absent from the source data, including losses associated with a saving failure, and were not evaluated by QC. High-frequency contamination (n = 12) comprises 11 records coded as high frequency and one originally coded as an amplitude issue subsequently attributed to high-frequency contamination. QC exclusions were channel-specific; no complete available experimental trial was excluded solely because of QC failure. Model observations are aggregated statistical observations rather than independent participants or trials. Each eligible coherence muscle-pair observation contributes one value in each of four frequency bands. QS = quiet sitting; F8 = figure-eight riding; SNG = stop-and-go riding; QC = quality control.
